# Enhancing Syntactic Complexity in L2 Chinese Writing: Effects of Form-Focused Instruction on the Chinese Topic Chain

**DOI:** 10.3389/fpsyg.2022.843789

**Published:** 2022-04-04

**Authors:** Jieyu Zhou, Chan Lü

**Affiliations:** Department of Asian Languages and Literature, University of Washington, Seattle, WA, United States

**Keywords:** syntactic complexity, topic chain, second language writing, form focused instruction, Chinese as a second language (CSL)

## Abstract

Syntactic complexity, as one aspect of the Complexity, Accuracy and Fluency (CAF) model, is integral to the writing ability of second language (L2) learners. Previous research found that T-unit-based measures of syntactic complexity in writing tasks of English as a second language (ESL) learners increased with more instruction, yet it remains unclear whether the same can be observed in Chinese as a second language (CSL) learners. To fill this gap, this study compared the development of syntactic complexity of a training group (*N* = 64) and a control group (*N* = 51), both composed of CSL students in a first-year Chinese course at a university in North America. While participants in the control group only participated in the regular course, the training group received an additional 10-week, explicit form-focused instruction (FFI) on the Chinese *topic chain*. Results on the posttest and delayed posttest show that the FFI on the topic chain had a positive and durable effect on the participants’ syntactic complexity; instructional intensity and feedback type may have influenced its effectiveness and durability. Characteristics of measures and task complexity may have affected the observation of syntactic complexity.

## Introduction

The development of writing abilities in a second language is critical in that it “is increasingly perceived as a central mechanism via which language competencies…perhaps must be acquired (Norris and Manchón, 2012, p. 221). To date, the triad model of CAF – complexity, accuracy and fluency – has been widely used for the assessment of second language writing abilities (Wolf-Quintero et al., 1998; Housen et al., 2012). Complexity, as the youngest dimension in the model, is the most complex and least understood dimension (Housen and Kuiken, 2009). At the syntactic level, complexity refers to the range and the sophistication of grammatical resources exhibited in language production (Ortega, 2015).

Previous studies investigating L2 written syntactic complexity have validated different measures of syntactic complexity (Wolf-Quintero et al., 1998; Lu, 2011; Kyle and Crossley, 2018) and used these measures to investigate the L2 writing development with and without pedagogical intervention (Ortega, 2003, 2015). Studies have found that syntactic complexity of L2 writing increases with more instruction (Mazgutova and Kormos, 2015; Vyatkina et al., 2015). However, currently available studies primarily focused on learners of English as a second language (ESL, Polio, 2017), leaving syntactic complexity in other languages like Chinese under-explored. More importantly, the measures and indicators for syntactic complexity in English may not necessarily be applicable or appropriate for measuring that in Chinese as a second language (CSL, Jin, 2007; Yu, 2021). Additionally, it is unclear whether syntactic complexity of Chinese, whether measured in oral or written discourse, can be enhanced by explicit instruction (Yu, 2019).

Therefore, guided by Norris and Ortega’s (2009) proposal for an organic approach to investigate syntactic complexity in instructed second language acquisition (SLA), as an exploration, this study attempts to examine if CSL learners’ syntactic complexity, based on current, validated measures for CSL learners proposed by Yu (2016, 2021), can be enhanced through a 10-week researcher-designed form-focused instruction.

## Literature Review

### Topic Chain in Measuring Syntactic Complexity in Chinese

Syntactic complexity is a multidimensional construct that can be measured through multiple scales (Norris and Ortega, 2009; Lu, 2011). Like English, Chinese syntactic complexity can be measured on global, clausal, and phrasal levels (Pan, 2018). However, since syntactic complexity entails features of language components and their composing mechanisms, the measurements of syntactic complexity used for one language may not be equally applicable to another.

Li and Thompson (1976) categorized English as a subject-prominent language whose sentences are formed with a structure of subject-predicate. In other words, an English sentence has one subject which is generally in the form of a noun or noun phrase (NP), and one predicate of which verb or verb phrase (VP) is the essential part. In contrast, Chinese is regarded as a topic-prominent language whose structure is better characterized as topic-comment (Chao, 1968; Li and Thompson, 1976; Chu, 1998). What distinguishes a topic from a subject is its thematic continuity and semantic “aboutness” in the discourse rather than a grammatical relationship with the rest of the clause (Chao, 1968; Givón, 1992). Given this character, a topic in Chinese can be not only a noun/NP but also a verb/VP or adjective; the comment is not limited to a verb/VP like in English but can also be a noun/NP. Therefore, the following sentence is grammatical in Chinese:

a. Wǒ shí suì. 我十岁。
  [I ten year old].
  I’m 10 years old.

The second difference between English and Chinese is that in English, the subject is usually stated to achieve a subject-predicate constituency, whereas Chinese is a pro-drop language whose subject or topic can be omitted when understood in the context (Huang, 1989). Therefore, when successive English clauses/sentences share one same subject, their subject positions need to be filled by either the full form of the subject, pronouns or demonstratives. However, in Chinese, when successive topic-comment structures share the same topic, the topic can be overtly stated only once and represented with zero anaphora in the rest of the positions, thus resulting in a topic chain (Tsao, 1979, 1990). Because the topic chain is constituted with multiple topic-comment structures despite the overtness of the topics, and the topic-comment structures are actually clauses (Tsao, 1990; Chu, 1998), the topic chain has a supra-clause character. In other words, the overt topic of the topic chain dominates successive clauses. Li (2004b) identified 10 types of topic chains, the most simple and typical form of which is one with overt topic in the first clause, just like the example provided here:

b. Wǒ jiào Xiǎohóng, jīnnián shí suì, zài shàng xiǎo xué. 我_i_叫小红, Ø_i_今年十岁, Ø_i_在上小学。
  [I_*i*_ call Xiaohong, Ø_*i*_ this year 10 year old, Ø_*i*_ ZAI-progressive marker attend primary school]
  I’m called Xiaohong. I’m 10 years old this year. I’m attending primary school.

It is proposed that topic chains are the basic and the most frequently used structure to construct discourse in Chinese, a discourse-oriented language (Tsao, 1979). Further, the order of the clauses in a topic chain follows the sequential order of events (Tai, 1985), therefore, complex sentences in Chinese can be understood as topic chains (Yu, 2021). English complex sentences, on the other hand, are realized through subordination of clauses, on which the measure of English syntactic complexity is based (Hunt, 1965).

Based on clausal subordination, Hunt (1965, 1970) developed a T-unit-based system of indices to measure the English syntactic complexity, which has been extensively used in a wide variety of languages (Cooper, 1976; Larsen-Freeman, 1978, 1983; Henry, 1996; Iwashita, 2006). A T-unit is defined as one independent clause plus, however, many subordinate clauses there are that are attached to the independent clause (Hunt, 1970). Although initially proposed to measure the syntactic complexity in L1 English (Hunt, 1965, 1970), T-unit related measurements such as T-unit length, error-free T-unit length, percentage of error-free T-unit, clauses per T-unit were used to assess the syntactic complexity of various Indo-European languages as L2s, and were proven sound measurements in L2 development (Scott and Tucker, 1974; Larsen-Freeman, 1978; Wolf-Quintero et al., 1998). However, due to the different constructions of complex sentences, it is argued that Chinese syntactic complexity should be measured based on topic chains, instead of Hunt’s T-unit system (Jin, 2007; Yu, 2021).

### Syntactic Complexity Development in Chinese as a Second Language

Studies have used both specific measurements and general measurements to investigate Chinese L2 syntactic complexity. Using specific measurements, studies have focused on the development of a collection of syntactic forms that are specific to Chinese (Han and Feng, 2017; Yang and Zhao, 2018). For example, the *ba*-construction, passive structure marked with *bei*, comparison sentences marked with *bi*, VOV 得 (de), etc. However, since studies covered different structures and employed different methods to calculate the complexity, their results are not always comparable. In addition, since the development of these syntactic forms are insignificant either across groups or across time according to the studies, and different syntactic forms usually have different developmental trajectories, the specific scale measurements, like Wolf-Quintero et al. (1998) suggested, are not the best indices to describe the syntactic complexity due to a lack of generalizability.

Studies using general measurements have utilized a T-unit system or topic-chain-based measurements. Drawing on the above-mentioned T-unit analysis, Jiang (2013) and An (2015) both found T-unit length sensitive in discriminating between the writings of higher-level Chinese L2 learners, but not so in discriminating between learners of lower language levels (Jiang, 2013). However, in Jin’s (2007) study measuring three levels of learners from intermediate to high levels and a group of native speakers, neither T-unit length, clause per T-unit nor T-unit per sentence effectively and significantly increased with language level. In fact, in her study, all three indices in native speakers’ language decreased. In other words, using the T-unit measurement, L2 Chinese learners were assessed to be utilizing more complex language than Chinese native speakers. In contrast, all indices based on the topic chain, mean length (character) of topic chain, clause per topic chain, and zero anaphoras per topic chain discriminated against each language level well in Jin’s (2007) study. A replication study (Wu, 2016) assessing novice, intermediate and advanced level learners and native Chinese speakers found the same results; namely, all of the topic chain measurements and none of the T-unit indices discriminated between the writings of the four language levels. Based on the findings mentioned above, it is clear that although subordination does exist in Chinese, subordination-based measurements like the T-unit system may not be the most sensitive measure in Chinese syntactic complexity. Following Norris and Ortega’s (2009) proposal for measuring complexity multi-dimensionally, we argue that topic-chain-based measurement should be more seriously considered in CSL research.

In light of the results in Jin (2007); Wu (2016), and Yu (2021) proposed an organic measure of Chinese syntactic complexity based on the topic chain, which resolved two remaining questions in Jin (2007): the relationship between topic chains and non-topic chain clauses, and the boundary of topic chains. In her analysis, Yu (2021) proposed a terminable Topic-Comment unit (TC-unit) and a single TC-unit as the basic operationalizable units capturing syntactic complexity of Chinese in the supra-clausal and clausal levels, respectively. “A terminable TC-unit is a supra-clausal-level unit that takes the form of a topic chain, and a single TC-unit is one clausal or subclausal level unit in Chinese” (Yu, 2021, pp. 9-10). A terminable TC-unit is a simple terminable TC-unit when it only contains one independent single TC-unit, or one topic-comment structure; it is a complex terminable TC-unit when it contains two or more dependent single TC-unit whose shared topic overtly appears once and represented with zero anaphora or coreferential zero in the remaining positions. Example *c* below is a complex terminable TC-unit consisting of two single TC-units, whereas Example *d* are two simple terminable TC-units each containing one single TC-unit.

c. Wǒ jiào Xiǎohóng, jīnnián shí suì. 我_i_叫小红, Ø_i_今年十岁。
  [I_*i*_ call Xiaohong, Ø_*i*_ this year 10 year old]
  I’m called Xiaohong. I’m 10 this year.
d. Wǒ jiào Xiǎohóng, wo jīnnián shí suì. 我叫小红, 我今年十岁。
  [I call Xiaohong. I this year 10 year old.]
  I’m called Xiaohong. I’m ten this year.

Further, Yu (2021) validated four measures of Chinese syntactic complexity in both written and spoken production of L1 and L2 Chinese speakers: (1) mean length of terminable TC-unit (MLTTCU), (2) complex terminable TC-unit in all terminable TC-units (CTTCU/ATTCU), (3) mean length of single TC unit (MLSTCU), and (4) single TC-units per terminable TC-unit (STCU/TTCU). The former two were considered as a global level measure and the latter two clausal level measures. Her results showed that participants with higher Chinese proficiency produced longer terminable TC-units consisting of more dependent single TC-units. Specifically, MLTTCU was the most effective indicator of spoken Chinese syntactic complexity and the combination of MLTTCU and STCU/TTCU was the most effective indicator of written Chinese syntactic complexity.

### Form-Focused Instruction, Topic Chain, and Syntactic Complexity in Chinese as a Second Language

The topic chain is one of the Chinese discourse features that may be subtle and opaque in nature to learners whose first languages do not employ such a feature. Given that learners allocate a varied level of attention to different layers of language (Talmy, 2008), they were found to display a lack awareness toward Chinese-specific discourse features (e.g., Jin, 1994; Polio, 1994; Yuan, 1995; Liu, 2015; Lu, 2019). However, on the other hand, the topic chain appears in the very first lesson of first-year Chinese textbooks, yet it is rarely taught explicitly in class (Li, 2004a,^2006^). This fact naturally leads to the question of whether CSL learners’ syntactic complexity in writing can be enhanced through *form-focused instruction* (FFI, e.g., Collins and Ruivivar, 2020), by explicitly directing their attention to the language form (in this case, topic chain) through direct instruction integrated with activities focused on communicating in the target language.

To date, only a handful of studies have investigated the effectiveness of FFI in CSL focusing on vocabulary, phonology (tones), grammar, and pragmatics (Yuan, 2018). Among the studies on grammar, most of the research was conducted on some form of pedagogical treatment (e.g., consciousness raising activities) or task conditions (e.g., dictogloss task) combined with different participant grouping on discrete syntactic forms such as the aspect marker *le* (Yuan, 2010, 2012a,2014a,2014b).

Only one study explored the effect of explicit instruction on the syntactic complexity of L2 Chinese learners. Yu (2019) elicited oral production in a class of second-year L2 learners in a university setting (*N* = 12). In the first week, the researcher showed a video in English about the Chinese New Year, and immediately asked the students to retell the story based on the video, followed by a 20-min explicit instruction on Chinese syntactic complexity. The students were then asked to complete another retell task based on the earlier prompt. In the following 3 weeks, the researcher conducted a 5-min explicit instruction each week on the topic chain compositionality. In week 3, the same video was shown again, and students were asked to complete the third retell task. The four measures validated in Yu (2021) were employed to compare students’ oral production in the three retell tasks. The results showed that MLTTCU and STCU/TTCU in the second retell task were significantly greater than that in the first, suggesting the immediate impact of the explicit instruction on the learners’ oral syntactic complexity. However, all four measures in the third retell task did not differ from that in the first, indicating a diminished effect of the researcher-designed instruction.

The results in Yu (2019) only demonstrated the positive role of the explicit instruction in enhancing L2 learners’ syntactic complexity in the oral modality. However, it is unclear if improved syntactic complexity through oral training can necessarily and readily improve that in writing (e.g., Bulté and Housen, 2009). Therefore, whether syntactic complexity in writing can be enhanced through explicit instruction remains unclear. Additionally, the intensity of the instruction in Yu’s (2019) study was relatively low, and the only available resources to students were limited to the instructor’s 35-min lecture across 4 weeks. These factors might have contributed to the low durability of the instructional effects. Moreover, it is difficult to tease apart the effect of natural growth vs. instructional effect, since there was no control group in the study.

To summarize, syntactic complexity in English and in Chinese is measured in different ways based on their respective linguistic features. The topic chain has been found to be a much more sensitive measure of syntactic complexity in CSL research, different from the T-unit-based measures widely used in other languages. The paucity of research exploring the instructional effect of the topic chain in CSL propelled us to investigate the effect of explicit instruction on syntactic complexity. Through a researcher-designed 10-week FFI focusing on the Chinese topic chain, the current study investigated the following questions:

1. Does explicitly teaching students how topic chains are constructed improve their syntactic complexity in writing tasks?
2. Does the explicit instruction have any delayed effect on the participants’ performance in writing tasks?

## Methodology

### Participants

Participants were recruited from a first-year Chinese course from a Chinese program at a large public university in the United States. Sixty-four out of the 115 students in the first-year course volunteered to participate and were considered as the training group (*N* = 64; 35 female and 29 male). They were given extra credit for finishing 80% of the tasks. The remaining fifty-one students who did not participate were considered as the control group (*N* = 51). None of the student had any prior experience learning the language nor the writing system, and were all considered true-beginners. Throughout the entire semester, the two groups followed the same instructional plan offered by the curriculum, while the training group received 10 weeks of explicit FFI on the Chinese topic chain and completed weekly writing assignments (12 in total across 10 weeks) in addition, at the end of the quarter, both the training group and the control group participated in a posttest, and a delayed posttest 2 weeks later.

Since this study lasted 10 weeks, individual-level random attrition (Pan and Zhan, 2020) – participants dropping out before the end of the study for a variety of reasons – was observed. Since individual attrition occured along the 10-week study, and the number of participants for each task was provided in Table 3 below. For the current study, incomplete datasets were not included in the final analysis.

**TABLE 1:** A coding sample of free writing task (ID: 101).

	STCU	TTCU	CTTCU	TTCUL
我_i_姓高 [I_*i*_ surname Gao] My family name is Gao	1	1	1	3
Ø_i_叫高文中 [Ø_*i*_ call Gao Wenzhong] My name is Gao Wenzhong	1			4
Ø_i_是美国人。 [Ø_*i*_ be American] I’m an American	1			4
我是学生。 [I be student] I’m a student	1	1		4
Total	4	2	1	15

*STCU, single TC-unit; TTCU, terminable TC-unit; CTTCU, complex terminable TC-unit; TTCUL, length of terminable TC-unit.*

**TABLE 2:** Calculated scores for the coding sample (ID: 101) on free writing task.

Task	MLTTCU	CTTCU/TTCU	MLSTCU	STCU/TTCU
FWT	7.5( = 15/2)	0.5( = 1/2)	3.75( = 15/4)	2( = 4/2)

*FWT, free writing task; MLTTCU, mean length of terminable TC-unit; CTTCU/TTCU, complex terminable TC-unit in all terminable TC-units; MLSTCU, mean length of single TC-unit; STCU/TTCU, single TC-units per terminable TC-unit.*

**TABLE 3:** Three stages of the assignments completed by the training group.

Task	Stage 1 (N)	Stage 2 (N)	Stage 3 (N)
DT	1st (63), 2nd (61)	3rd (58), 4th (53)	5th (43)
RRT	1st (50), 2nd (53), 3rd (52)	4th (50), 5th (46), 6th (45)	7th (43), 8th (33)
FWT	1st (63), 2nd (61), 3rd (57)	4th (55), 5th (51), 6th (49)	7th (46), 8th (43), 9th (43), 10th (33)

*N, number of participants; DT, deletion task; RRT, rearrange and rewrite task; FWT, free writing task.*

### Instructional Materials

#### Instructional Videos

All of the instruction and writing assignments were delivered through the online platform Moodle. There were six instructional videos in total, designed and narrated by the first author, and ranged from 3 to 5 min long. The six instructional videos were based on the content of the first-semester Chinese curriculum, and their release date synchronized with the teaching schedule of the course. Following Yu; Yu’s (2016; 2019) summary of the rules of thumb and boundaries of the topic chain, the videos included the form, function, and appropriate contexts for the usage of the Chinese topic chain. The contents of the videos are as follows:

Video 1: Introduction to the function and form of topic chains, or why and how to use them.
Video 2: Three conditions where topic chains were not applicable, or the chain boundaries.
Videos 3–5: Each video included detailed explanations and examples of one condition mentioned in Video 2.
Video 6: Review of the content in the previous five videos and comments on frequent errors found in the participants’ assignments.

Two written assignments (detailed below) were given for each instructional video; participants’ responses to these written assignments were collected as data points for later analysis. On average, the participants have spent 180 min completing all the required tasks according to our log on Moodle^1^.

#### Assignments

Each assignment contained two questions using three types of tasks (1) delete repetitive elements from a sentence Deletion Task (DT); (2) rearrange sentences into an appropriate order and rewrite them into a cohesive paragraph rearrange and rewrite task (RRT), (3) write a coherent paragraph(s) according to the given instructions free writing task (FWT). DT was the least challenging and appeared in the assignments the earliest because it only required students to recognize and delete repeated topics. RRT appeared after DT because the ability of grouping sentences based on “aboutness” was needed in addition to deleting repeated topics. FWT came last because it required students to initiate topic chains without scaffolding, and was thus considered most challenging. All assignments were untimed but students were instructed to complete them within about 15 min. Through weekly emails to all participants in the training group, the instructor summarized and explained common errors found in the participants’ assignments with grammatical rules and examples.

#### Posttest and Delayed Posttest

There was a 2-week interval between the posttest and the delayed posttest. Each posttest included one RRT and one FWT of the same format and difficulty level. The RRT consisted of 12-13 sentences, and the FWT was based on a set of pictures which depicted a series of events involving multiple characters, therefore requiring the participants to pay attention to topics, and their changes, in constructing clauses.

### Scoring

For DT, since the target noun or pronoun can be either correctly or incorrectly deleted, we assigned one (1) point to every correctly deleted element and zero (0) point to an incorrectly deleted element and then calculated the total raw score. For this measure, 20% of the data was double-coded by the two researchers, and complete agreement (100%) was reached. Participants’ responses on RRT and FWT were based on the following four validated measurements of Chinese syntactic complexity (Yu, 2021), namely, MLSTCU and STCU/TTCU on clausal level; MLTTCU and CTTCU/TTCU on global level.

First, the researcher segmented sentences into STCUs and identified TTCUs. Second, the total number of STCU, TTCU, CTTCU, and the length of TTCU, STCU, and CTTCU in character in each participant’s written production in each task were counted either manually or using formulas in Excel. Character-based length measures rather than word-based measures were chosen because the two measures do not make any difference in presenting L2 Chinese writing development, and the former is more reliable for length calculation (Jiang, 2013). Third, scores of each of the four measures: Mean Length of terminable TC-unit (MLTTCU), Complex terminable TC-unit/all terminable TC-units (CTTCU/TTCU), Mean length of single TC-unit (MLSTCU), and Single TC-units per terminable TC-unit (STCU/TTCU), were calculated. The length was measured in characters.

A coding sample is provided in Table 1. It codes an entry on a FWT from participant 101: Wǒ xìng Gāo, jiào Gāo Wénzhōng, shì Měiguó rén. Wǒ shì xuésheng. 我姓高, 叫高文中, 是美国人。我是学生。 [I_*i*_ surname Gao, Ø_*i*_ call Gao Wenzhong, Ø_*i*_ be American. I be student.] My family name is Gao. My name is Gao Wenzhong. I’m an American. I’m a student. There are four *single TC-units*, therefore four STCUs. TTCUs were segmented based on overt topics. Since there are two overt topics “我” (*I*), there are two TTCUs. Because there is only one topic chain that contains more than one single TC-unit, the number of CTTCU is one. And since TTCU includes both single STCU and CTTCU, the lengths of the TTCUs are 15 characters, 11 characters in the CTTCU and 4 characters in STCU, respectively. Table 2 shows an example of the calculations based on the example provided in Table 1.

### Procedures

This project was designed based on the syllabus of the 10-week Chinese course and started in the second week of the quarter. In the first week of the quarter, all students enrolled in First Year Chinese were invited to join this researcher-designed training on Moodle. From Week 2 to Week 8, the training group engaged in the training program as we described above, in addition to their daily 50-min class; students in the control group only participated in the daily 50-min class offered by the Chinese program. In Week 10, a posttest was given to all students enrolled in First Year Chinese, including students in both the training group and the control group. After a 2-week winter break, a delayed posttest was delivered to the same group of students.

During the 10-week study, five Deletion Tasks (DT), eight Rearrange and Rewrite Tasks (RRT), and ten Free Writing Tasks (FWT) were assigned to students in the training group. Since the three tasks all contained multiple data points, we grouped them into Stage 1 (S1), Stage 2 (S2), and Stage 3 (S3), as shown in Table 3, to better demonstrate the developmental trajectories of the training group in all three tasks.

The number of participants in the training group decreased gradually over time due to individual-level random attrition (Pan and Zhan, 2020). In addition, the number of participants in the training group who completed each task in the same assignment differed, because some of them finished the tasks selectively. Therefore, we only included data for those who completed a substantial portion (at least 66%) of each task during the 10-week training considering sufficient participation is required to guarantee learning. The detailed numbers of participants for each task are listed in Table 4.

**TABLE 4:** Mean values of each measure in all the tasks.

Task	Stage	N	MLTTCU	MLSTCU	CTTCU/TTCU	STCU/TTCU	Correct
**Training group in the 12 written assignments**							
DT	S1	53	–	–	–	–	1.79
	S2	49	–	–	–	–	1.23
	S3	43	–	–	–	–	2.37
RRT	S1	38	10.66	7.80	0.36	1.42	–
	S2	38	12.80	8.64	0.47	1.51	–
	S3	33	17.31	9.37	0.46	1.86	–
FWT	S1	45	7.16	4.06	0.65	1.80	–
	S2	43	10.47	7.59	0.45	1.45	–
	S3	30	11.60	8.24	0.23	1.43	–
**Training group in the posttest (comparing with control group in posttest)**							
RRT		41	10.73	7.51	0.38	1.44	–
FWT		43	10.56	7.55	0.26	1.42	–
**Control group in the posttest**							
RRT		31	9.66	7.78	0.22	1.44	–
FWT		25	9.28	7.61	0.15	1.23	–
**Training group in the posttest (comparing with delayed posttest)**							
RRT		32	10.75	7.50	0.39	1.44	–
FWT		31	10.50	7.46	0.26	1.42	–
**Training group in the delayed posttest**							
RRT		32	9.21	6.61	0.32	1.40	–
FWT		31	12.42	8.44	0.25	1.49	–

*MLTTCU, mean length of terminable TC-unit; MLSTCU, mean length of single TC-unit; CTTCU/TTCU, complex terminable TC-unit in all terminable TC-units; STCU/TTCU, single TC-units per terminable TC-unit; correct, scores of deletion task; RRT, rearrange and rewrite task; FWT, free writing task; DT, deletion task; S1, S2, S3, stage 1, stage 2, stage 3; N: number of participants.*

## Results

Table 4 shows the mean scores of each measure in all tasks completed by both the training group and the control group. In terms of the assignment results of the training group during the 10-week study, the score of DT decreased from S1 to S2 before a spur in S3. Meanwhile MLTTCU, MLSTCU and STCU/TTCU of RRT all grew from S1 to S3 while CTTCU/TTCU saw a moderate dipping in S3 after an increase from S1-S2. Although both length measures MLTTCU and MLSTCU of FWT increased from S1 to S3, its two ratio measures CTTCU/TTCU and STCU/TTCU decreased along the way.

Comparing the post-test results of the training group and the control group, it was found that in both the RRT and FWT, the training group scored higher in MLTTCU, CTTCU/TTCU but slightly lower in MLSTCU; the two groups’ scores on STCU/TTCU were identical.

In terms of the performance of the training group in the posttest and the delayed posttest, all four measures of RRT were lower in the delayed posttest than in the posttest. In FWT, on the contrary, MLTTCU, MLSTCU and STCU/TTCU were higher and CTTCU/TTCU was lower in the delayed posttest than in the posttest.

### Research Question 1: Does Explicitly Teaching Students How Topic Chains Are Constructed Improve Their Syntactic Complexity in Writing?

To answer Research Question 1, the developmental trajectories of syntactic complexity of the training group’s scores on the three tasks were calculated and compared, and the posttest results of this group and the control group were compared. Syntactic complexity was measured by the scores of the three tasks, namely, Deletion Task, Rearrange and Rewrite Task, and Free Writing Task.

Non-parametric Friedman tests were used to detect differences in the performance of the training group among the three stages on each task because our data did not meet the normality assumption of the repeated measures ANOVA. In addition, post hoc analysis with Wilcoxon signed-rank tests was further conducted with a Bonferroni correction (corrected *p* = 0.017) to compare each pair of stages. Results are listed in Table 5.

**TABLE 5:** Z and *p* values of Wilcoxon signed-rank tests of the training group on all tasks in the written assignments.

	MLTTCU		MLSTCU		CTTCU/TTCU		STCU/TTCU		Correct	
	*Z*	*p*	*Z*	*p*	*Z*	*p*	*Z*	*p*	*Z*	*p*
**Deletion task**										
S2-S1	–	–	–	–	–	–	–	–	−5.276	0.000
S3-S1	–	–	–	–	–	–	–	–	−1.959	0.025
S3-S2	–	–	–	–	–	–	–	–	−3.969	0.000
**Rearrange and rewrite task (RRT)**										
S2-S1	−4.693	0.000	−4.898	0.000	−3.190	0.000	−2.085	0.000	–	–
S3-S1	−4.741	0.000	−4.741	0.000	−2.026	0.021	−3.960	0.000	–	–
S3-S2	−3.752	0.000	−3.752	0.000	−0.681	0.252	−2.877	0.001	–	–
**Free writing task (FWT)**										
S2-S1	−5.645	0.000	−5.645	0.000	−4.692	0.000	−5.351	0.000	–	–
S3-S1	−4.703	0.000	−4.703	0.000	−4.703	0.000	−4.552	0.000	–	–
S3-S2	−1.826	0.035	−2.955	0.001	−3.844	0.000	−0.985	0.168	−	–

*MLTTCU, mean length of terminable TC-unit; MLSTCU, mean length of single TC-unit; CTTCU/TTCU, complex terminable TC-unit in all terminable TC-units; STCU/TTCU, single TC-units per terminable TC-unit; Correct, scores of deletion task; S2-S1, the difference between stage 2 and stage 1; S3-S1, the difference between stage 3 and stage 1; S3-S2, the difference between stage 3 and stage 2.*

In terms of the Deletion Task, an overall significant difference between the three stages on the score of correct deletion was found, χ^2^(2) = 25.389, *p* = 0.000. *Post hoc* analysis found a significant decrease from S1 to S2 (*Z* = −5.276, *p* = 0.000), and a significant increase from S2 to S3 (*Z* = −3.969, *p* = 0.000). However, the difference between S1 and S3 (*Z* = −1.959, *p* = 0.025) was not significant. This suggested that the training group’s ability to correctly delete redundant topics and create topic chains (complex TTCUs) remained at the same level throughout the three stages although fluctuation occurred in the process, indicating a stable performance of the training group with the Deletion Task.

On the Rearrange and Rewrite Task, an overall significant difference (increase) between the three stages on measures MLSTCU [χ^2^(2) = 36.741, *p* = 0.000] and STCU/TTCU [χ^2^(2) = 16.280, *p* = 0.000] was found, indicating a development in the syntactic complexity on the clausal level. On the other hand, an overall significant difference (increase) between the three stages was found on MLTTCU [χ^2^(2) = 40.963, *p* = 0.000] but not on CTTCU/TTCU [χ^2^(2) = 4.168, *p* = 0.125], yet a post hoc analysis revealed that the score of CTTCU/TTCU significantly grew from S1 to S2, indicating that the syntactic complexity also increased significantly at the global level.

On the Free Writing Task, an overall significant increase was found on the two length measures MLTTCU [χ^2^(2) = 40.231, *p* = 0.000], MLSTCU [χ^2^(2) = 43.923, *p* = 0.000], and an overall significant decrease was found on the two ratio measures CTTCU/TTCU [χ^2^(2) = 42.117, *p* = 0.000] and STCU/TTCU [χ^2^(2) = 29.176, *p* = 0.000]. This suggests that as their learning progressed, students in the training group produced longer but fewer topic chains. Although the ratio measures decreased, the increased length measures of MLSTCU and MLTTCU still suggest a growing syntactic complexity on both clausal and global levels, respectively. The implication of this finding will be further discussed in the next section.

In addition, due to the unequal sample sizes, a Welch’s t-test was conducted to compare the posttest performance of the training group and the control group. The results are listed in Table 6.

**TABLE 6:** Mean and standard deviation of the training and the control group on posttest in Welch’s *t*-test.

Measure	Experimental group		Control group	
	*M*	*SD*	*M*	*SD*
**Free writing task**				
MLTTCU	10.56	1.52	9.28	1.90
MLSTCU	7.55	1.32	7.61	1.17
CTTCU/TTCU	0.26	0.12	0.15	0.15
STCU/TTCU	1.42	0.23	1.23	0.23
**Rearrange and rewrite task**				
MLTTCU	10.73	1.62	9.66	1.57
MLSTCU	7.51	0.41	7.78	0.52
CTTCU/TTCU	0.38	0.18	0.22	0.21
STCU/TTCU	1.44	0.25	1.25	0.25

*M, mean value; SD, standard deviation; MLTTCU, mean length of terminable TC-unit; MLSTCU, mean length of single TC-unit; CTTCU/TTCU, complex terminable TC-unit in all terminable TC-units; STCU/TTCU, single TC-units per terminable TC-unit; correct, scores of deletion task.*

On the Rearrange and Rewrite Task, the training group had significantly higher scores than the control group in MLTTCU, *t*(65.780) = 2.830, *p* = 0.006, CTTCU/TTCU, *t*(58.943) = 3.222, *p* = 0.002 and STCU/TTCU, *t*(64.447) = 3.108, *p* = 0.003; yet its score on MLSTCU, *t*(55.646) = −2.346, *p* = 0.023, was significantly lower than the control group. On the Free Writing Task, participants in the training group scored significantly higher than the control group in the measures MLTTCU, *t*(41.868) = 2.871, *p* = 0.006, CTTCU/TTCU, *t*(42.725) = 3.121, *p* = 0.003, and STCU/TTCU, *t*(50.831) = 3.423, *p* = 0.001. The two groups did not differ on MLSTCU, *t*(55.358) = −0.181, *p* = 0.857. The results above indicated that the training group had a significantly greater syntactic complexity than the control group on both clausal level and global level in both Free Writing Task and Rearrange and Rewrite Task. The decreased MLSTCU in RRT will be further discussed in the next section.

In sum, our findings suggested that the syntactic complexity of the training group grew significantly during the 10-week period and better performance was observed in the training group than the control group based on the results of the posttest, at both clausal and the global levels.

### Research Question 2: Does the Explicit Instruction Have Any Delayed Effect on the Participants’ Performance?

To answer Research Question 2, performance on the posttest and the delayed posttest of the training group were compared through a Wilcoxon signed-rank test because our data did not meet the normality assumption of the dependent *t*-test.

Results of the Wilcoxon signed-rank test showed that on the Rearrange and Rewrite Task, the scores of the measures CTTCU/TTCU (*Z* = −1.831, *p* = 0.067) and STCU/TTCU (*Z* = −1.211, *p* = 0.226) did not differ in the delayed posttest from the posttest, yet the scores of MLTTCU (*Z* = −4.338, *p* = 0.000) and MLSTCU (*Z* = −4.937, *p* = 0.000) decreased significantly in the delayed posttest, indicating an undermined delayed training effect on this task.

With the Free Writing Task, the scores of MLTTCU (*Z* = −2.972, *p* = 0.003) and MLSTCU (*Z* = −4.086, *p* = 0.000) increased significantly in the delayed posttest while the scores of CTTCU/TTCU (*Z* = −0.625, *p* = 0.532) and STCU/TTCU (*Z* = −0.660, *p* = 0.509) did not have a significant difference between the posttest and the delayed posttest. This suggested a durable training effect on this task.

To summarize, the 10-week explicit FFI did have a delayed effect on the participants’ performance, especially on the Free Writing Task.

## Discussion

Our study examined whether CSL learners’ syntactic complexity can be enhanced by a 10-week researcher-designed FFI on constructing the Chinese topic chain. Our overall findings suggest that the 10-week FFI on constructing the topic chain had a positive and durable effect on learners’ syntactic complexity on both the clausal and global levels. Our discussion here highlights the important roles of instructional intensity and feedback type in influencing the effectiveness and durability of FFI, and the characteristics of measures and task complexity in affecting the observation of syntactic complexity.

### Unequal Sensitivity and Asynchronous, Non-linear Development of the Measures

The first research question focuses on the immediate effects of the 10-week FFI on syntactic complexity. The training group’s development over time, and the control group’s performance of the posttest showed that the instruction had an immediate effect in improving the participants’ Chinese syntactic complexity. However, different measures showed unequal sensitivity under different comparing conditions: when performances of the same group (in our case the training group) were compared across time, syntactic complexity was better reflected by length measures MLTTCU and MLSTCU, whereas when two different groups were compared (training vs. control group), ratios measures CTTCU/TTCU and STCU/TTCU and the global-level length measure MLTTCU demonstrate the syntactic complexity better.

In addition, opposite to the training group’s positive development and better performance in general, some measures showed decreased or less competitive results during the training period and in the posttest respectively, indicating that the four measures did not necessarily develop in the same pattern or synchronously, and their development was not linear. In the 10-week training period, the ratio measures CTTCU/TTCU and STCU/TTCU in the Free Writing Task decreased significantly. This is somewhat different from Yu’s (2021) finding that the two measures positively predicted the syntactic complexity in the Free Writing Task. However, this may not necessarily suggest that our training did not have any effect, or had a negative impact on learners’ syntactic complexity in the Free Writing Task. The decreased performance on one task is certainly possible, especially when the 10-week instruction is rather brief compared to the time one would spend on learning a language. One possible explanation to the decreased trend is that with the natural growth of the training groups’ language performance, they produced a greater number of sentences in the Free Writing Task, resulting in a larger TTCU as the denominator, thus the two ratio measures CTTCU/TTCU and STCU/TTCU shrank. Meanwhile, in the posttest, the training group scored significantly lower than the control group in the clausal-level length measure MLSTCU in the Rearrange and Rewrite Task. Although this result seems to undermine the training effect, MLSTCU was found to be not predictive of written syntactic complexity (Yu, 2021). Despite its insignificance, the decreased trend of MLSTCU could be due to the tendency that the repetitive subjects/topics were more likely to be deleted by the training group than by the control group, resulting in topic chains (CTTCU) in greater length and number. This can be supported by the significantly greater ratio measures (CTTCU/TTCU and STCU/TTCU) and global-level length measure (MLTTCU) in the training group, compared to the control group. Such complementary findings in the current study are in line with Norris and Ortega’s (2009) argument that complexity should be measured multidimensionally, and the idea that increased clausal complexification can be achieved via coordination; the findings are also consistent with Yu (2021), which found that MLSTCU was not predictive of written syntactic complexity.

The second research question investigated the delayed effect of the 10-week FFI on syntactic complexity. In the delayed posttest, the training group’s performance was maintained well on all four measures of the Free Writing Task, yet in the Rearrange and Rewrite Task, it was only maintained on the ratio measures but not the length measures. This result indicates that on one hand, our 10-week FFI, in contrast to that in Yu (2019), had a delayed training effect on the participants; on the other hand, the delayed training effect was unevenly reflected by the two writing tasks.

### The Influence of Instructional Intensity and Feedback Type

Regarding the positive delayed training effect, despite the modality difference, we believe that the instructive intensity and the available learning resources contributed greatly to the different results between Yu (2019) and the present study. In Yu (2019), the only resource for students to learn about the topic chain or syntactic complexity was the instructor’s 35-min instruction in total, whereas in the present study, in addition to the 24-min video instructions, students also spent 180 min on average to practice what was taught through the 12 assignments of three task types.

Additionally, the instructor-provided feedback in the two studies were different. Yu (2019) provided corrective feedback (CF) to individual students in class orally. In contrast, the feedback provided in the weekly emails and the last instructional video in the present study can be categorized as written and oral metalinguistic explanation (ME), respectively (Sheen, 2007; Shintani and Ellis, 2013). For example, students tended to overgeneralize topic omission, illustrated in the following example (Table 7). In the email feedback addressed to all participants, the learned rule was listed first, *repetitive subjects that are in the same chain should be on the same position, or to the same side of the verb*, followed by the ME and correction.

**TABLE 7:** An example of metalinguistic explanation provided to participants in the study.

	Examples and explanation
Error	*这是我妈妈, 是医生。 “This is my mom, is doctor”
Full form sentence	这_subj_是_verb_我妈妈_obj_。我妈妈_subj_是_verb_医生_obj_。 “This is my mom. My mom is a doctor”
ME	我妈妈_obj_ and 我妈妈_subj_ are repetitive, but not on the same side of the verb, therefore it cannot be omitted, but could be replaced by a pronoun
Correct sentence	这是我妈妈。她是医生。 “This is my mom. She is a doctor”

*ME, metalinguistic explanation.*

The ME provided in the current study, exemplified in Table 7, may have directed the learners’ awareness to the understanding level (Schmidt, 1995), rather than just at the noticing level. Our explanation is also supported by findings from previous studies comparing ME and other types of feedback in classroom settings. Previous studies have found advantages of ME, either oral or written, over CF (Carroll and Swain, 1993; Shintani and Ellis, 2013) and ME in conjunction with CF over CF alone (Bitchener et al., 2005; Bitchener, 2008; Bitchener and Knoch, 2009), in their immediate effects on ESL learners’ acquisition of English articles and dative verbs, as well as their durable effects (Sheen, 2007; Bozorgian and Yazdani, 2021). In contrast, immediate feedback provided as CF was ineffective in developing learners’ awareness of the rule as the learners only developed consciousness at the noticing level (Sheen, 2007; Shintani and Ellis, 2013).

Similarly, research on the effects of ME on CSL learners’ acquisition of Chinese-specific grammatical features has found that ME had significant immediate and durable effects in both oral and written modalities (*wh*-questions, and classifiers, Wu, 2019), and the effects were stronger for lower-proficiency level learners (Li, 2014). Additionally, ME was more effective on the rule-governed syntactic structures *wh*-question and perfective aspect *le* than the more salient, exemplar-based lexical items, classifiers. Li (2014) used selective attention (Gass, 1997) to explain the imbalanced ME effects and stated that ME helped direct the limited attention/resources of L2 learners, especially beginners, to semantically opaque structures. The Chinese topic chain structure can also be regarded as a rule-governed structure that requires learners’ selective attention, and our findings contributed to our understanding of how metalinguistic explanation, embedded in form-focused instruction, can contribute to the development of novice learners’ syntactic complexity through writing tasks. Given that the learners’ language system is feedback-sensitive, it would be interesting for future studies to explore if intermediate level CSL learners, who are still learning Chinese-specific grammatical patterns, can also benefit from ME.

### Task Complexity and Attention

The uneven delayed effect of the two tasks may be explained by task complexity. The Free Writing Task in the current study can be considered cognitively more complex than the Rearrange and Rewrite Task along the resource-directing dimension, according to the criteria for task complexity proposed by Robinson (2001, 2003, 2011, 2015). Meanwhile, the same hypothesis claims that compared to simpler tasks, increasing the cognitive complexity of task demands on the resource-directing level leads to more complex production, and greater depth of processing and long-term retention of input. Although empirical studies generated mixed evidence based on this hypothesis, there are studies showing that writing tasks of higher cognitive complexity due to increased number of elements and increased level of reasoning lead to greater syntactic complexity (Abrams, 2019; Rahimi, 2019; Golparvar and Rashidi, 2021). As discussed above, in addition to deleting repetitive topics and combining sentences as Rearrange and Rewrite Task, the Free Writing Task also required an extra element of reasoning – forming basic sentences – which made it more cognitively complex. Therefore, it might be reasonable to conclude that task complexity of the Free Writing Task was a potential contributing factor to the imbalanced durability of the 10-week instruction. However, existing evidence supporting the effect of cognitively complex tasks on learners’ written syntactic complexity is mostly from European languages as target languages. To our best knowledge, only one study (Yuan, 2012b) investigated the effects of tasks with different cognitive complexity on syntactic complexity in the CSL context. Yuan (2012b) found that CSL learners’ written syntactic complexity did not change significantly depending on the provision of the outline of a composition, while their written fluency increased due to the decreased cognition load. Although this finding does not contribute to the interpretation of our results directly, it lends supportive evidence to Robinson’s Cognition Hypothesis. However, more studies are needed to further discuss the influence of task complexity on CSL learners’ syntactic complexity.

### Pedagogical Implication

Though the topic chain is an important feature of the Chinese language (Tsao, 1979, 1990; Li, 2004b), and that researchers have studied its difficulty levels (Li, 2006), its acquisition patterns (Jin, 2007), and made pedagogical proposals (Li, 2006; Jin, 2007), but “[S]urprisingly, few studies have taken steps further to test the recommendations in research.” (Yuan, 2018, p. 42). This current study, therefore, serves as one of the first studies to empirically test whether explicitly teaching the topic chain through form-focused instruction to true beginners can lead to increased syntactic complexity in their writing. We provided empirical evidence to show such an endeavor is meaningful, and our findings suggest that the explicit instruction of the topic chain should be emphasized in CSL classrooms. In addition, we proved the effectiveness and the necessity of metalinguistic explanation in improving CSL learners’ syntactic complexity. Widely used textbooks for CSL learners in North America, such as *Integrated Chinese* (Liu et al., 2017), do include practices about the topic chain, yet such components usually appear at the end of each chapter, a place least likely to be noticed by learners. Explicit instruction of the topic chain is usually not included in Chinese instructors’ teaching plans (Li, 2006), despite its importance for developing syntactic complexity. Our findings suggest that the topic chain cannot be easily mastered through self-learning. As a rule-governed syntactic structure, its mastery requires metalinguistic explanation to direct beginning learners’ limited attention to such a semantically opaque structure.

## Conclusion

This study examined whether CSL learners’ written syntactic complexity can be enhanced by a 10-week researcher-designed form-focused instruction on constructing the Chinese topic chain. In general, immediate and sustainable training effects with statistical significance were found through the training group’s development in the training period; the training group displayed better performance than the control group in the posttest and well maintained their performance in the delayed posttest. Meanwhile, the findings point to the importance of instructional intensity and types of feedback in influencing the effectiveness and durability of form-focused instruction, as well as characteristics of measures and task complexity in affecting the observation of syntactic complexity.

### Limitations

The study has several limitations. First, the study only examined the performance of one single proficiency level, and the sample size was relatively small and unequally distributed in different tasks due to attrition. However, as students’ participation was strictly voluntary, it was difficult to ensure uniformity across the 10 weeks for all participants. Second, the study did not randomly assign students to the training vs. control groups because the Institutional Review Board of the institution where the study took place required the study to be implemented on a voluntary basis. Therefore, though a randomized design would be ideal, it was not feasible at the time. Thirdly, since the training was conducted online, it was hard to know whether students really invested themselves in the learning activities or only finished them quickly based on their existing knowledge in order to earn the extra credits, especially when the 10-week training can be weary and make participants gradually lose interest. Relatedly, the study did not address how individual differences at the learner level can affect the effectiveness of the training. In addition, due to COVID19, a quarter before the onset of this project, all instruction at the university abruptly went virtual. Therefore, during the implementation of this project, there was no in-person communication between the instructor and the learners; there could have been other unknown factors in the participants’ lives which may have influenced their performance in this study and their academic performance in general.

While encouraging findings were found in the present study, further research is needed to replicate or refine the present study with larger and more stable sample size, ideally with random assignment, under a more controllable teaching environment, as results found under one condition or context may not necessarily generalize under another (e.g., Rogers and Cheung, 2021). Further, the effects of the form-focused instruction across different proficiency levels and across modalities deserve further exploration. Additionally, future studies can also investigate how long the syntactic complexity can be maintained.

## Data Availability Statement

The datasets presented in this study can be found in online repositories. The names of the repository/repositories and accession number(s) can be found below: Open Science Framework: https://osf.io/h7zgj/.

## Ethics Statement

The studies involving human participants were reviewed and approved by University of Washington. The patients/participants provided their written informed consent to participate in this study.

## Author Contributions

CL provided conceptualization and design of the study. JZ and CL designed and reviewed all the instructional and test materials used in the study. JZ collected the data, and conducted the statistical analysis. Both authors wrote the first draft of the manuscript and contributed to manuscript revision, read, and approved the submitted version.

## Conflict of Interest

The authors declare that the research was conducted in the absence of any commercial or financial relationships that could be construed as a potential conflict of interest.

## Publisher’s Note

All claims expressed in this article are solely those of the authors and do not necessarily represent those of their affiliated organizations, or those of the publisher, the editors and the reviewers. Any product that may be evaluated in this article, or claim that may be made by its manufacturer, is not guaranteed or endorsed by the publisher.

## Footnotes

1 All materials used in the study can be viewed here: https://osf.io/h7zgj/?view_only=0a2f4447cf95424f9f1c3fbba5b61b12.

## References

[B1] Abrams Z. I. (2019). The effects of integrated writing on linguistic complexity in L2 writing and task-complexity. *System* 81 110–121. 10.1016/j.system.2019.01.009

[B2] An F. (2015). Butong shuiping CSL xuexizhe zuowen liuchangxing jufa fuzadu he zhunquexing fenxi: Yixiang jiyu T danwei celiangfa de yanjiu [Analysis of fluency, grammatical complexity and accuracy of CSL writing: a study based on T-unit analysis]. *Yuyan Jiaoxue Yanjiu [Lang. Teach. Linguist. Stud.]* 3 11–20.

[B3] Bitchener J. (2008). Evidence in support of written corrective feedback. *J. Second Lang. Writ.* 17 102–118. 10.1016/j.jslw.2007.11.004

[B4] Bitchener J. Knoch U. (2009). The contribution of written corrective feedback to language development: a ten-month investigation. *Appl. Linguist.* 31 193–214. 10.1093/applin/amp016

[B5] Bitchener J. Young S. Cameron D. (2005). The effect of different types of corrective feedback on ESL student writing. *J. Second Lang. Writ.* 14 191–205. 10.1016/j.jslw.2005.08.001

[B6] Bozorgian H. Yazdani A. (2021). Direct written corrective feedback with metalinguistic explanation: investigating language analytic ability. *Iran. J. Lang. Teach. Res.* 9 65–85.

[B7] Bulté B. Housen A. (2009). “The development of lexical proficiency in L2 speaking and writing tasks by Dutch-speaking learners of French in Brussels,” in *Paper Presented in the colloquium ‘Tasks across modalities’, Task-based Language Teaching Conference*, (Lancaster).

[B8] Carroll S. Swain M. (1993). Explicit and implicit negative feedback: an empirical study of the learning of linguistic generalizations. *Stud. Second Lang. Acquis.* 15 357–386. 10.1017/s0272263100012158

[B9] Chao Y. R. (1968). *A Grammar of Spoken Chinese.* Berkeley, CA: University of California Press.

[B10] Chu C. C. H. (1998). *A Discourse Grammar of Mandarin Chinese (Vol.6).* New York, NY: Peter Lang Pub Incorporated.

[B11] Collins L. Ruivivar J. (2020). “Form–focused instruction,” in *The Concise Encyclopedia of Applied Linguistics*, ed. Chapelle C. A. (Hoboken, NJ: John Wiley and Sons Inc). 10.1002/9781405198431.wbeal0432.pub2

[B12] Cooper T. C. (1976). Measuring written syntactic patterns of second language learners of German. *J. Educ. Res.* 69 176–183. 10.1080/00220671.1976.10884868

[B13] Gass S. (1997). *Input, Interaction, and the Second Language Learner.* Mahwah, NJ: Erlbaum.

[B14] Givón T. (1992). *English Grammar: A Function-Based Introduction (Vol. 2).* Amsterdam: John Benjamins. 10.1075/z.engram2

[B15] Golparvar S. E. Rashidi F. (2021). The effect of task complexity on integrated writing performance: the case of multiple-text source-based writing. *System* 99:102524. 10.1016/j.system.2021.102524

[B16] Han X. Feng L. (2017). Hanyu kouyu jufa fuzadu fazhan ceping zhong jizhun zhibiao de yingyong fangfa yanjiu [Application of benchmark indexes in development research of syntactic complexity in Chinese L2 speaking]. *Shijie Hanyu Jiaoxue [Chin. Teach. World]* 31 542–559.

[B17] Henry K. (1996). Early L2 writing development: a study of autobiographical essays by university-level students of Russian. *Modern Lang. J.* 80 309–326. 10.1111/j.1540-4781.1996.tb01613.x

[B18] Housen A. Kuiken F. (2009). Complexity, accuracy, and fluency in second language acquisition. *Appl. Linguist.* 30 461–473. 10.1093/applin/amp048

[B19] Housen A. Kuiken F. Vedder I. (2012). *Dimensions of L2 Performance and Proficiency: Complexity, Accuracy and Fluency in SLA (Vol. 32).* Amsterdam: John Benjamins Publishing. 10.1075/lllt.32

[B20] Huang C. T. J. (1989). “Pro-drop in Chinese: a generalized control theory,” in *The Null Subject Parameter*, Vol. 15 eds Jaeggli O. A. Safir K. J. (Dordrecht: Springer), 185–214. 10.1007/978-94-009-2540-3_6

[B21] Hunt K. W. (1965). Grammatical Structures Written at Three Grade Levels (Report No. 3). National Council of Teachers of English Research Report. Available onine at: https://files.eric.ed.gov/fulltext/ED113735.pdf (accessed December 18, 2021).

[B22] Hunt K. W. (1970). “Recent measures in syntactic development,” in *Readings in Applied Transformational Grammar*, ed. Lester M. (New York, NY: Holt, Rinehart and Winston, Inc), 187–200.

[B23] Iwashita N. (2006). Syntactic complexity measures and their relation to oral proficiency in Japanese as a foreign language. *Lang. Assess. Q.* 3 151–169. 10.1207/s15434311laq0302_4

[B24] Jiang W. (2013). Measurements of development in L2 written production: the case of Chinese L2. *Appl. Linguist.* 34 1–24. 10.1093/applin/ams019

[B25] Jin H. G. (1994). Topic-prominence and subject-prominence in L2 acquisition: evidence of English-to-Chinese typological transfer. *Lang. Learn.* 44 101–122.

[B26] Jin H. G. (2007). Syntactic maturity in second language writings: a case of Chinese as a foreign language (CFL). *J. Chin. Lang. Teach. Assoc.* 42 27–55.

[B27] Kyle K. Crossley S. A. (2018). Measuring syntactic complexity in L2 writing using fine-grained clausal and phrasal indices. *Modern Lang. J.* 102 333–349. 10.1111/modl.12468

[B28] Larsen-Freeman D. (1978). An ESL index of development. *Tesol Q.* 12 439–448. 10.2307/3586142

[B29] Larsen-Freeman D. (1983). “Assessing global second language proficiency,” in *Classroom Oriented Research in Second Language Acquisition*, eds Seliger H. W. Long M. H. (Rowley, MA: Newbury House Publishers, Inc), 287–304.

[B30] Li C. N. Thompson S. (1976). “Subject and topic: a new typology of language,” in *Subject and Topic*, ed. Li C. N. (New York, NY: Academic Press), 457–489.

[B31] Li S. (2014). The interface between feedback type, L2 proficiency, and the nature of the linguistic target. *Lang. Teach. Res.* 18 373–396. 10.1177/1362168813510384

[B32] Li W. (2004b). Topic chains in Chinese discourse. *Discourse Process.* 37 25–45. 10.1207/s15326950dp3701_2

[B33] Li W. (2004a). The discourse perspective in teaching Chinese grammar. *J. Chin. Lang. Teach. Assoc.* 39 25–44.

[B34] Li W. (2006). Ba huatilian naru hanyu yufa jiaoxue tixi – hanyu yupian tedian zai waiyu jiaoxue zhong de tixian [Incorporating topic chains into pedagogical grammar of Chinese]. *J. Chin. Lang. Teach. Assoc.* 41 36–56.

[B35] Liu F. H. (2015). Acquiring topic structures in Mandarin Chinese. *Chin. Second Lang. Res.* 4 1–21. 10.1016/j.cub.2020.01.059 32142700 PMC7141976

[B36] Liu Y. Yao T. C. Bi N. P. Ge L. Shi Y. (2017). *Integrated Chinese: Zhongwen Ting Shuo du Xie.* Boston, MA: Cheng and Tsui.

[B37] Lu X. (2011). A corpus-based evaluation of syntactic complexity measures as indices of college-level ESL writers’ language development. *Tesol Q.* 45 36–62. 10.5054/tq.2011.240859 24853049

[B38] Lu Y. (2019). L2 distribution of Chinese connectives: towards a comprehensive understanding of a discourse grammar. *Second Lang. Res.* 35 557–586. 10.1177/0267658318791662

[B39] Mazgutova D. Kormos J. (2015). Syntactic and lexical development in an intensive English for Academic Purposes programme. *J. Second Lang. Writ.* 29 3–15. 10.1016/j.jslw.2015.06.004

[B40] Norris J. M. Manchón R. M. (2012). “Investigating L2 writing development from multiple perspectives: issues in theory and research,” in *L2 Writing Development: Multiple Perspectives*, ed. Manchón R. M. (Boston, MA: De Gruyter Mouton), 221–244. 10.1515/9781934078303.221

[B41] Norris J. M. Ortega L. (2009). Towards an organic approach to investigating CAF in instructed SLA: the case of complexity. *Appl. Linguist.* 30 555–578. 10.1093/applin/amp044

[B42] Ortega L. (2003). Syntactic complexity measures and their relationship to L2 proficiency: a research synthesis of college-level L2 writing. *Appl. Linguist.* 24 492–518. 10.1093/applin/24.4.492

[B43] Ortega L. (2015). Syntactic complexity in L2 writing: progress and expansion. *J. Second Lang. Writ.* 29 82–94. 10.1016/j.jslw.2015.06.008

[B44] Pan X. (2018). *Investigating the Development of Syntactic Complexity in L2 Chinese Writing* Unpublished doctoral dissertation. Iowa City, IA: University of Iowa.

[B45] Pan Y. Zhan P. (2020). The impact of sample attrition on longitudinal learning diagnosis: a prolog. *Front. Psychol.* 11:1051. 10.3389/fpsyg.2020.01051 32655428 PMC7325952

[B46] Polio C. (1994). Nonnative speakers’ use of nominal classifiers in Mandarin Chinese. *J. Chin. Lang. Teach. Assoc.* 29 51–66.

[B47] Polio C. (2017). Second language writing development: a research agenda. *Lang. Teach.* 50 261–275. 10.1017/s0261444817000015

[B48] Rahimi M. (2019). Effects of increasing the degree of reasoning and the number of elements on L2 argumentative writing. *Lang. Teach. Res.* 23 633–654. 10.1177/1362168818761465

[B49] Robinson P. (2001). Task complexity, task difficulty, and task production: exploring interactions in a componential framework. *Appl. Linguist.* 22 27–57. 10.1093/applin/22.1.27

[B50] Robinson P. (2003). The cognition hypothesis, task design, and adult task-based language learning. *Second Lang. Stud.* 21 45–105.

[B51] Robinson P. (2011). *Second Language Task Complexity: Researching the Cognition Hypothesis of Language Learning and Performance (Vol. 2).* Amsterdam: John Benjamins Publishing. 10.1075/tblt.2

[B52] Robinson P. (2015). “The Cognition Hypothesis, second language task demands, and the SSARC model of pedagogic task sequencing,” in *Domains and Directions in the Development of TBLT*, Vol. 8 ed. Bygate M. (New York, NY: John Benjamins Publishing Company), 87–121. 10.1075/tblt.8.04rob

[B53] Rogers J. Cheung A. (2021). Does it matter when you review? Input spacing, ecological validity, and the learning of l2 vocabulary. *Stud. Second Lang. Acquis.* 43 1138–1156. 10.1017/S0272263120000236

[B54] Schmidt R. (1995). *Attention and Awareness in Foreign Language Learning Second Language Teaching and Curriculum Center.* Honolulu, HI: University of Hawaii.

[B55] Scott M. Tucker G. (1974). Error analysis and English-language strategies of Arab students. *Lang. Learn.* 24 69–97. 10.1111/j.1467-1770.1974.tb00236.x

[B56] Sheen Y. (2007). The effect of focused written corrective feedback and language aptitude on ESL learners’ acquisition of articles. *Tesol Q.* 41 255–283. 10.1002/j.1545-7249.2007.tb00059.x

[B57] Shintani N. Ellis R. (2013). The comparative effect of direct written corrective feedback and metalinguistic explanation on learners’ explicit and implicit knowledge of the English indefinite article. *J. Second Lang. Writ.* 22 286–306. 10.1016/j.jslw.2013.03.011

[B58] Tai J. H.-Y. (1985). “Temporal sequence and word order in Chinese,” in *Iconicity in Syntax*, ed. Haiman J. (New York, NY: John Benjamins Publishing Company), 49–72. 10.1075/tsl.6.04tai

[B59] Talmy L. (2008). “Aspects of attention in language,” in *Handbook of Cognitive Linguistics and Second Language Acquisition*, eds Robinson P. Nick E. C. (New York, NY: Routledge), 27–38.

[B60] Tsao F. (1979). *A Functional Study of Topic in Chinese: The First Step Towards Discourse Analysis (Vol. 3).* Taipei: Student Book Company.

[B61] Tsao F. (1990). *Sentence and Clause Structure in Chinese: A Functional Perspective (Vol. 14).* Taipei: Student Book Company.

[B62] Vyatkina N. Hirschmann H. Golcher F. (2015). Syntactic modification at early stages of L2 German writing development: a longitudinal learner corpus study. *J. Second Lang. Writ.* 29 28–50. 10.1016/j.jslw.2015.06.006

[B63] Wolf-Quintero K. Inagaki S. Kim H. Y. (1998). *Second Language Development in Writing: Measures of Fluency, Accuracy, and Complexity, Second Language Teaching and Curriculum Center.* Honolulu, HI: University of Hawaii.

[B64] Wu J. (2016). Yingyu muyuzhe hanyu shumianyu jufa fuzaxing yanjiu [The grammatical complexity in English native speakers’ Chinese writing]. *Yuyan Jiaoxue Yanjiu [Lang. Teach. Linguist. Stud.]* 4 27–35.

[B76] Wu Y. (2019). “Differential effects of recasts and metalinguistic prompts on the acquisition of Chinese Wh-questions and classifiers,” in *Classroom Research on Chinese as a Second Language*, eds Yuan F. Li S. (New York, NY: Routledge), 45–76.

[B65] Yang L. Zhao Z. (2018). Profiling L2 writing development: the case of CFL learners in intermediate classes. *Chin. Second Lang. Res.* 7 221–247. 10.1515/caslar-2018-0009

[B66] Yu Q. (2016). *Defining and Assessing Chinese Syntactic Complexity via TC–Units* Unpublished doctoral dissertation. Honolulu, HI: University of Hawaii at Manoa.

[B67] Yu Q. (2019). “Effects of explicit instruction on Chinese syntactic complexity in repeated story–retelling tasks,” in *Classroom Research on Chinese as a Second Language*, eds Yuan F. Li S. (New York, NY: Routledge), 100–127. 10.4324/9780203709740-5

[B68] Yu Q. (2021). An organic syntactic complexity measure for the Chinese language: the TC-Unit. *Appl. Linguist.* 42 60–92. 10.1093/applin/amz064

[B69] Yuan B. (1995). Acquisition of base-generated topics by English-speaking learners of Chinese. *Lang. Learn.* 45 567–603. 10.1111/j.1467-1770.1995.tb00455.x

[B70] Yuan F. (2010). Impacts of task conditions on learners’ output of L2 Chinese. *J. Chin. Lang. Teach. Assoc.* 45 67–88.

[B71] Yuan F. (2012a). Effects of consciousness on “LE” in L2 Chinese: a pilot study in a classroom setting. *J. Chin. Lang. Teach. Assoc.* 47 65–90.

[B72] Yuan F. (2012b). Renwu tiaojian hexiezuo xingshi dui hanyu eryu xiezuo zhiliang he shuliang de yingxiang [The impacts of task conditions and writing modes on the output quality and quantity in writing of Chinese as a second language]. *Taiwan Huayu Jiaoxue Yanjiu [Taiwan Chin. Teach. Res.]* 1 33–50.

[B73] Yuan F. (2014a). “Focused dictogloss, peer collaboration, and guided reconstruction: a case of time expressions in L2 Chinese,” in *Advances in Chinese as a Second Language: Acquisition and Processing*, ed. Jiang N. (Newcastle: Cambridge Scholars Publishing), 133–151.

[B74] Yuan F. (2014b). Pushed-output, peer collaboration, and L2 Chinese learning: product and process. *Huayuwen Jiaoxue Yanjiu [J. Chin. Lang. Teach.]* 3 33–60.

[B75] Yuan F. (2018). “Form–focused instruction and task–based language teaching in Chinese as a second language,” in *The Routledge Handbook of Chinese Second Language Acquisition*, ed. Ke C. (London: Routledge), 415–431. 10.4324/9781315670706-19

